# Stimuli-responsive and core cross-linked micelles developed by NiCCo-PISA of helical poly(aryl isocyanide)s†

**DOI:** 10.1039/d2py00397j

**Published:** 2022-06-13

**Authors:** Sètuhn Jimaja, Spyridon Varlas, Jeffrey C. Foster, Daniel Taton, Andrew P. Dove, Rachel K. O'Reilly

**Affiliations:** Department of Chemistry, University of Warwick Coventry CV4 7AL UK; School of Chemistry, University of Birmingham Edgbaston B15 2TT UK a.dove@bham.ac.uk r.oreilly@bham.ac.uk; Laboratoire de Chimie des Polymères Organiques, Université de Bordeaux/CNRS École Nationale Supérieure de Chimie, de Biologie & de Physique 33607 Cedex Pessac France

## Abstract

We report the synthesis of redox- and pH-sensitive block copolymer micelles that contain chiral cores composed of helical poly(aryl isocyanide)s. Pentafluorophenyl (PFP) ester-containing micelles synthesised *via* nickel-catalysed coordination polymerisation-induced self-assembly (NiCCo-PISA) of helical poly(aryl isocyanide) amphiphilic diblock copolymers are modified post-polymerisation with various diamines to introduce cross-links and/or achieve stimulus-sensitive nanostructures. The successful introduction of the diamines is confirmed by Fourier-transform infrared spectroscopy (FT-IR), while the stabilisation effect of the cross-linking is explored by dynamic light scattering (DLS). The retention of the helicity of the core-forming polymer block is verified by circular dichroism (CD) spectroscopy and the stimuli-responsiveness of the nanoparticles towards a reducing agent (l-glutathione, GSH) and pH is evaluated by following the change in the size of the nanoparticles by DLS. These stimuli-responsive nanoparticles could find use in applications such as drug delivery, nanosensors or biological imaging.

## Abstract 2

Spherical micelles with a helical core synthesised by NiCCo-PISA are functionalised with different cross-linkers to make stimulus-sensitive nanostructures.

## Graphical abstract

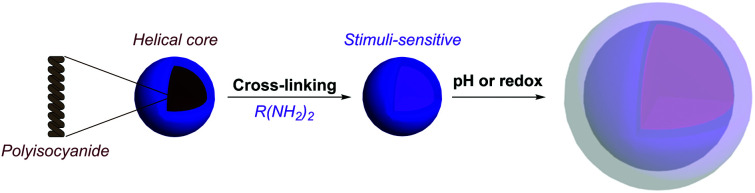

## 1. Introduction

Smart nanomaterials that undergo alteration when subjected to a particular stimulus have received significant levels of attention.^1–4^ The design and development of these stimuli-responsive materials are of interest for applications such as drug delivery,^5–8^ biological imaging,^7,9,10^ chiroptical materials^11^ and sensing.^12–14^ A wide variety of different triggers have been developed, including pH,^12,15–20^ temperature,^19,21–25^ light,^26–31^ magnetic field,^32–34^ CO_2_,^35^ glucose,^36–38^ β-cyclodextrin,^39^ ions,^14,40,41^ electric potential,^42^ and redox potential.^16,43–46^ Systems with a range of different responses can be achieved as a result of an alteration in the solubility of the nanoparticles (NPs), such as changes in size^19,24,31^ and morphology,^47–49^ light emission,^11,13^ and permeability.^27,28,50,51^ Additionally, reactive cross-linkers can be employed to impart responsiveness to the resulting three-dimensional polymeric network. Cross-linked nanoparticles are of interest for their improved stability, which allows the conservation of the nanoparticle morphology under changes to their solvation state and/or concentration.

A potential application of stimuli-responsive self-assembled NPs is their triggered disassembly, usually intended for the design of drug delivery systems that necessitates a release of their cargo in a specific microenvironment, *e.g.* acidic or reducing conditions found in tumours. Cross-links that are cleaved under these conditions allow for a controlled stimulus-responsive disassembly. For example, cross-linkers containing disulfide bonds that are reactive towards thiols and reducing compounds,^43,45,52,53^ or acetals that can be cleaved under acidic conditions^16,54,55^ have been successfully employed.

Helical polymers that present a static structure display optical activity that leads them to play an important role in areas such as chiral recognition^27,56,57^ or catalysis.^58–62^ We hypothesised that the combination of helicity alongside stimuli-responsiveness could lead to materials of potential importance in the domain of asymmetric catalysis or drug-delivery. For instance, one could envision a chiral nanoreactor that would only react with one enantiomer of a racemic mixture, which would thus eliminate complex purification steps for on-demand delivery of compounds with high enantiopurity. Helical polymers, such as polyisocyanides, have been successfully employed as stimuli-responsive building blocks. In a noteworthy report, Wu and co-workers developed a dual-responsive nanomaterial that responded both to oxidation and pH changes able to release its cargo in a cell-like environment.^63,64^ Indeed, combining two triggers (oxidation and pH) increased the solubility of the core in water, causing the micelles to disassemble and release their cargo.^65^ However, the synthetic methodologies used to achieve stimuli-responsive NPs proved time- and resource-consuming and give NP dispersions with a relatively low content of polymer. An alternative method to readily prepare nanostructures is polymerisation-induced self-assembly (PISA), which is a versatile technique to achieve nano-objects at high solid concentrations using simple procedures.^66–71^ PISA is a one-pot self-assembly process that has been employed with various polymerisation techniques^71–74^ in a wide range of solvents.^75–78^ The adaptability of PISA and the ability to achieve a pure phase of the desired morphology in a predictable manner makes it ideal for development of new nanomaterials.^79–85^

Herein, we report the development of core-functionalised poly(aryl isocyanide) nanostructures synthesised by nickel-catalysed coordination polymerisation-induced self-assembly (NiCCo-PISA). Subsequent post-polymerisation modification (PPM) of pentafluorophenyl (PFP) activated ester units^43,86^ using a variety of primary diamines, enabled cross-linking of the core of the derived micelles. The latter cross-linking step provided stability and/or stimuli-responsive properties to the final nanostructures. The stimuli-responsiveness, and resultant disassembly, of the resulting cross-linked nanoparticles in a reducing environment or low pH was monitored by dynamic light-scattering (DLS). We think the combination of helicity and stimuli-responsiveness into nano-objects will open new avenues for delivery of chiral therapeutics. Moreover, the micelles as chiral platform developed by NiCCo-PISA were easily modified to introduce the wanted moiety, which paves the road to other applications such as enantioselective catalyst and circularly polarised light emitters.

## 2. Results and discussion

### 2.1 Cross-linking of NiCCo-PISA micelles

Building on the NiCCo-PISA-derived functionalisable micelles presented in our earlier works,^86,87^ the synthesis of stimulus-responsive nanostructures through a PPM methodology was envisioned. Namely, a functionalisable poly(PEG-ester aryl isocyanide)_20_-*block*-poly(menthyl-ester aryl isocyanide_15_-*co*-pentafluorophenyl-ester aryl isocyanide_15_), *i.e.* P(PAIC)_20_-*b*-P(MAIC_15_-*co*-FAIC_15_) or D50% (diblock polymer containing 50% of functionalisable core units), and its non-functionalisable counterpart, *i.e.* P(PAIC)_20_-*b*-P(MAIC_30_) or D0%, were synthesised *via* NiCCo-PISA in DMSO at 5 wt% solids content. These polyisocyanides were synthesised in earlier studies, which contain complete characterisation data for the polymers.^86,87^ Basic characterisation data, including molecular weight and dispersity, is summarized here (Table 1).

**Table 1:** Characterisation of the NiCCo-PISA block copolymer micelles before and after core cross-linking

Sample	*D*_DLS_ DMSO^a^ (nm)	*D*_DLS_ H_2_O^a^ (nm)	*D*_DLS_ THF^a^ (nm)	*D*_TEM_ b (nm)	Zeta pot. (mV)	CD_360_^c^ (mdeg)
D0%	20 (0.18)	58 (0.23)	—d	21 ± 7	−15 ± 5	14
D50%	20 (0.25)	140 (0.15)	—d	18 ± 4	+7 ± 7	8.7
D50% + HDA	20 (0.14)	100 (0.20)	28 (0.24)	15 ± 3	−1 ± 3	6.2
D50% + CA	19 (0.10)	180 (0.29)	26 (0.20)	16 ± 3	−13 ± 3	6.5
D50% + AEE	22 (0.15)	55 (0.18)	26 (0.16)	15 ± 3	−9 ± 9	8.9

a Hydrodynamic diameter measured by DLS at a 173° angle. PD is in parenthesis.

b Average diameter measured from dry-state TEM images from water suspension of NPs.

c CD signal at *λ* = 360 nm in THF.

d No assemblies in THF.

**Scheme 1:**
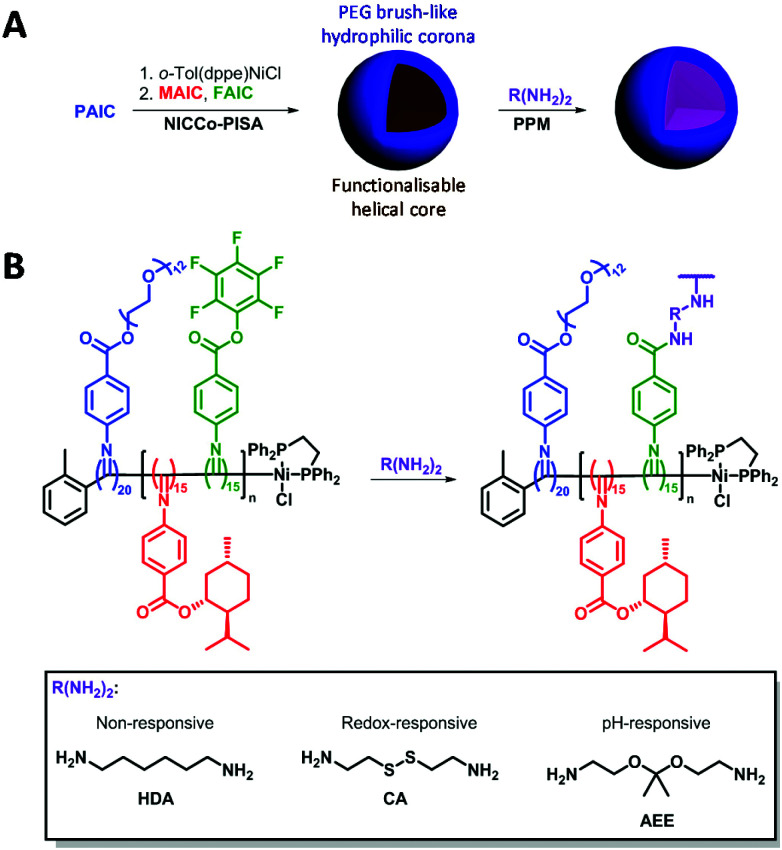
(A) Overview of the NiCCo-PISA of block copolymer micelles and post-polymerisation modification (PPM). (B) Detailed PPM procedure for the development of cross-linked and stimuli-responsive nanomaterials.

Here, direct cross-linking of the D50% micelles in DMSO was attempted in order to impart stability and stimulus-responsiveness to the nanostructures. Three cross-linkers were investigated: 1,6-hexanediamine (HDA) as a non-responsive cross-linker; cystamine (CA) that contains a redox-sensitive disulfide moiety and 2-[1-(2-amino-ethoxy)-1-methyl-ethoxy]-ethylamine (AEE) that bears a pH-sensitive acetal linker (Scheme 1). Redox and pH stimuli were selected as these are potentially useful for tumour targeting.^45,88–93^ Conditions from a previous report were used for the cross-linking experiments.^86^

**Fig. 1:**
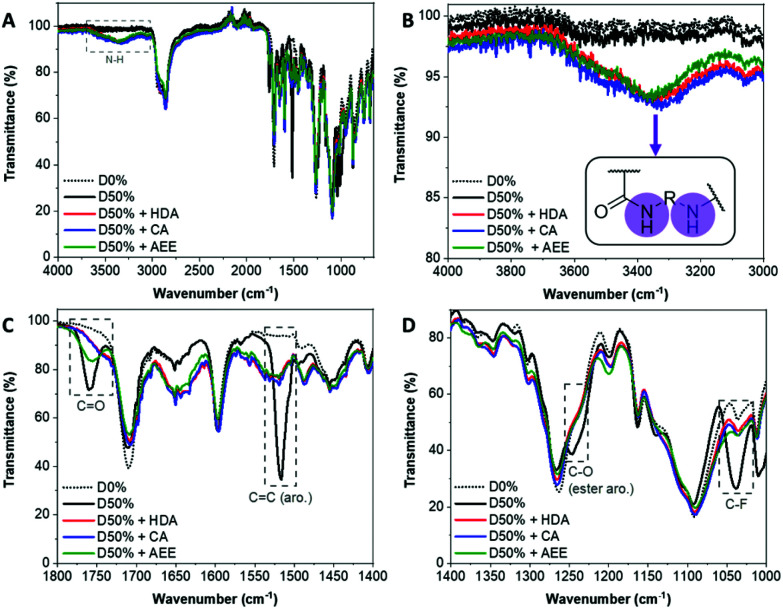
FT-IR spectra of the D0% (dotted black line) and D50% copolymer (black line) cross-linked with HDA (red line), CA (blue line) and AEE (green line). (A) Full spectrum. (B) Zoom in the 4000–3000 cm^−1^ region. (C) Zoom in the 1800–1400 cm^−1^ region. (D) Zoom in the 1400–1000 cm^−1^ region.

The NiCCo-PISA suspension of non-functionalised D50% NPs was reacted with 0.6 equivalents of each of the diamine cross-linkers for three days in DMSO at 50 °C. These PPM reactions were repeated using non-functional particles D0% (*i.e.* P(PAIC)_20_-*b*-P(MAIC_30_)), as controls. The reaction mixtures were dialysed against water to remove any unreacted diamines and an aliquot was freeze-dried prior to analysis. Analysis of the purified copolymer samples by FT-IR (Fig. 1) was used to determine the completion of the core cross-linking reaction in each case. The disappearance of three vibration signals indicated the absence of PFP esters: 1520 cm^−1^ for the C=C aromatic bond signal, 1240 cm^−1^ for the C–O ester bond and 1030 cm^−1^ for the C–F bond (Fig. 1C). Moreover, the signal from the C=O ester at 1755 cm^−1^ was replaced by a weaker and broader signal at 1750 cm^−1^, which originated from the newly formed amide bond (C=O stretching) (Fig. 1D). Finally, a new broad signal appeared at 3350 cm^−1^, which verified the formation of new amide N–H bonds (Fig. 1A and B). ^19^F NMR analysis of the cross-linked materials also showed the disappearance of the PFP group, which indicated that the reactions were highly efficient (Fig. S1†).

**Fig. 2:**
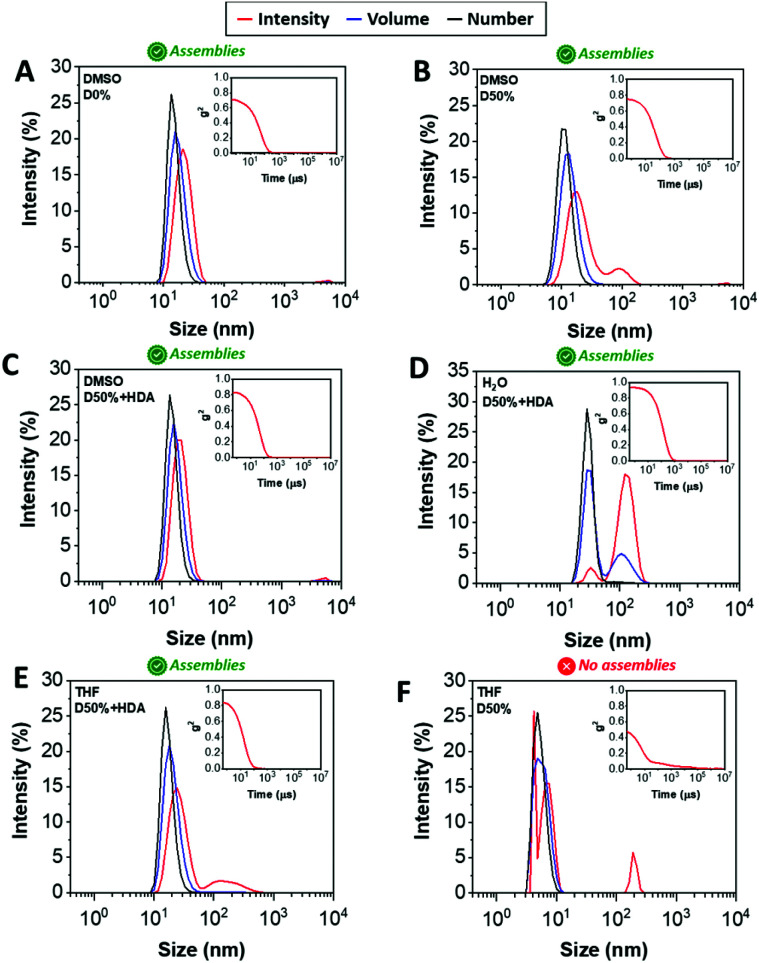
Size distributions of (A) D0%, (B) D50%, and (C) D50% + HDA in DMSO, (D) water, (E) THF, and (F) D50% in THF obtained by DLS. The intensity (red line), volume (blue line) and number (black line) distributions are displayed. The insets show the corresponding correlograms in each case.

DLS analysis of the nano-objects obtained after cross-linking indicated no major change in the NP size distribution, showing sizes around 20 nm and polydispersities (PDs) in line with the micelles prior to cross-linking (Fig. 2A–C, S3† and Table 1). These cross-linked nanostructures were subsequently transferred from DMSO to water by dialysis, which led to a change from transparent solutions in DMSO to opaque suspensions in aqueous media, for D0%, D50%, D50% + HDA and D50% + CA. Further analysis by DLS showed an increase in size of the particles from 20 nm in DMSO to 100–200 nm in water (Fig. 2D and S3†). In contrast, the size of D50% + AEE (55 nm) was found to be approximately the same to the originally determined value in DMSO. These larger apparent nano-object sizes could have been a consequence of the swelling of the NPs in water, their aggregation or a combination of these two effects. The possibility for the nano-objects to aggregate was investigated by determining the zeta-potential of the suspension of micelles in water, which was found to vary between +7 to −15 mV (Table 1). It thus appeared that the surface charges were too low to prevent aggregation *via* electrostatic repulsion, providing an explanation for the increase in size upon transferring the micelles to water.

One way to verify whether polymeric NPs are successfully stabilised by cross-linking is to transfer them into a good solvent for both constituent blocks. Non-cross-linked nano-objects would be expected to disassemble under such conditions, which is typically reflected by a reduced scattering intensity by DLS. In contrast, efficient cross-linking should lead to NPs showing approximately the same size to those collected in selective solvent (*i.e.* DMSO). The dialysed micelles were thus freeze-dried and re-solvated in THF, which proved to be a good solvent for both blocks. Analysis of these THF solutions by DLS confirmed the presence of nano-objects of similar dimensions to the NPs suspended in DMSO (Fig. 2E and S3†), while the non-cross-linked nano-objects D0% displayed no self-assembly (Fig. 2F). These results demonstrated that efficient cross-linking took place using the different diamines, HDA, CA and AEE, providing stabilising effect to the cross-linked cores.

The morphology of the cross-linked nano-objects suspended in water was then investigated by transmission electron microscopy (TEM). Spherical micelles with similar sizes to those obtained in DMSO were observed, which supported the hypothesis that large sizes as measured by DLS originated from local flocculation (Fig. S8†). The TEM images showed the presence of a population of individual micelles and revealed that the morphology of the spherical NPs did not change. For the isolated particles, the average diameters measured from the dry-state TEM images (upon counting >250 particles in each case) were 15–21 nm, slightly smaller than the size measured by DLS in DMSO. This can be explained by shrinkage of the micelles caused upon drying during TEM grid preparation.

**Fig. 3:**
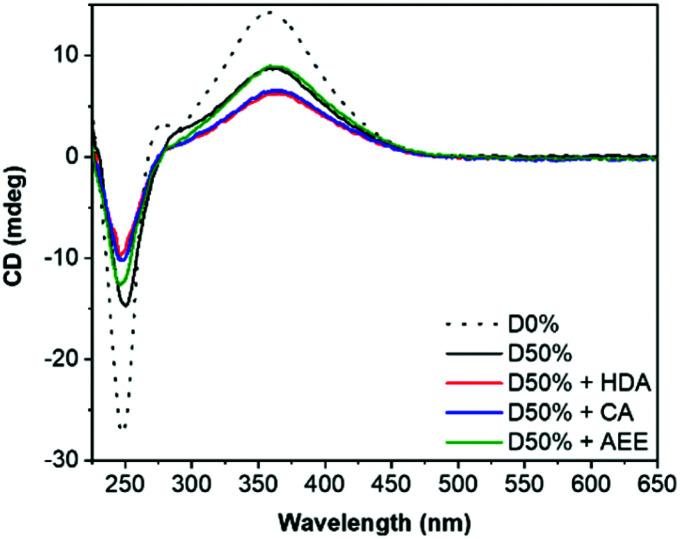
CD (THF, 0.5 mg mL^−1^) spectra of the unsubstituted D50% and its cross-linked counterparts. D0% is shown as reference.

Retention of the helicity of the micellar cores was investigated by circular dichroism (CD) spectroscopy of the cross-linked micelles in THF, to ensure solvation of both blocks at 0.5 mg mL^−1^. This CD analysis after cross-linking indicated a slight decrease of the CD signal at *λ* = 360 nm (CD_360_) for D50% + HDA and D50% + CA, from 8.7 mdeg for the unreacted copolymer to 6.2 mdeg and 6.5 mdeg, respectively, for the cross-linked micelles (Fig. 3). However, helicity was mainly retained (>70% compared to D50%) after cross-linking. No loss in helicity was reported for D50% + AEE. This is promising for applications that would leverage the helical core of the nano-objects. This also establishes that the core's helices could withstand the reaction conditions employed during the PPM step.

### 2.2 Redox- and pH-responsive NiCCo-PISA micelles

**Fig. 4:**
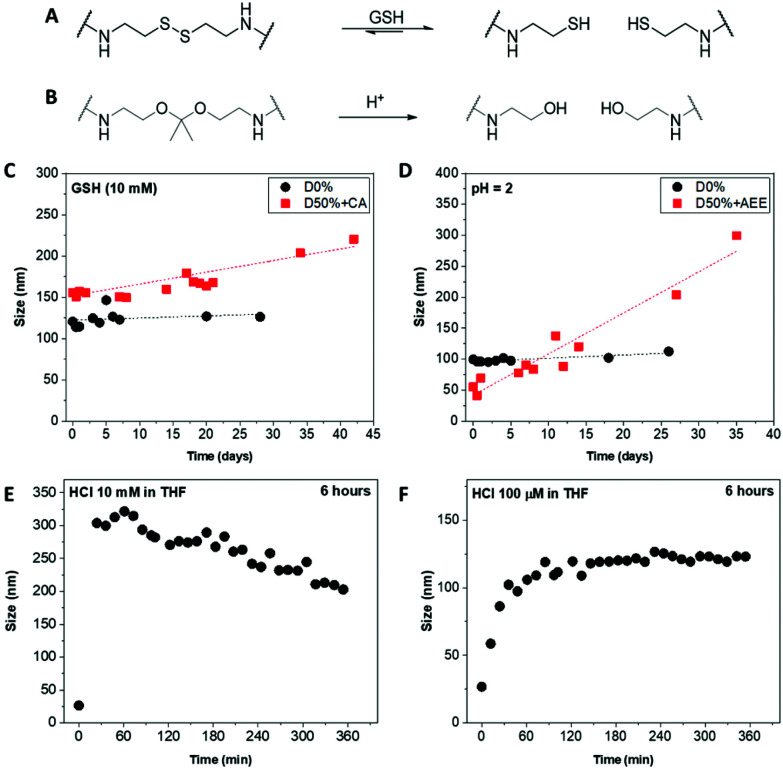
Scheme of (A) the GSH-triggered cleavage of the cystamine disulfide linkers and (B) the acid-triggered cleavage of the acetal linkers. Size evolution of (C) the CA-modified nanostructures upon treatment with GSH and the AEE-modified nanostructures at (D) pH = 2 in water, (E) 10 mM HCl and (F) 100 μM HCl in THF monitored by DLS.

Core-cross-linked micelles D50% + CA and D50% + AEE were expected to exhibit responsiveness towards reducing environment and low pH, respectively (Fig. 4A and B). The stimuli-responsive behaviour of D50% + CA and D50% + AEE NPs was thus investigated using DLS analysis. CA cross-linked NPs (D50% + CA) in water at a concentration of 0.2 mg mL^−1^ were subjected to l-glutathione (GSH) reducing agent at a concentration of 10 mM. The disulfide bond was expected to be cleaved at this [GSH], which would result in the formation of more solvophilic thiol moieties inside the core and change the assembly of the nano-objects. ^1^H NMR spectroscopy analysis of the cross-linked structures yielded broad signals with poor resolution (Fig. S2†). Moreover, TEM analysis was also not employed since analogous polyisocyanide nanoparticles exhibit inherent aggregation effects, which would obfuscate any changes to particle size.^86,87^ Instead, the size distribution of the NPs was monitored by DLS over a period of 4 weeks, in the presence of reducing agent. The redox-responsive NPs showed a size increase over this time period (Fig. 4C). This observed change in size can be explained by the gradual increase in the core's hydrophilicity, which led to a swelling of the NPs. Moreover, the cross-link density in the micelle cores slowly decreased, resulting in “looser” particles that can either swell or aggregate, or both. The increase in size was found to be progressive over a period of several days, which proved to be a slower process as compared to previously reported systems.^43,45^ This could be linked to a number of elements including the hydrophobicity of the core, aggregation of NPs and solubility. First, the high hydrophobicity of the NPs core – that contained l-menthyl side-chains – could slow down the access of the hydrophilic reducing agent.^94^ Secondly, the aggregation behaviour of the NPs might further limit the diffusion which might have delayed the disassembly of the systems into unimers and could explain the continuous increase in size. Also, the switch from stabilising disulfide bridges to thiol functionalities increased the exchange rate of the unimers, favouring micelle aggregation or rearrangement, which in turn led to the size increase observed for D50% + CA, from 155 nm to 220 nm. Finally, it was likely that the thiol-functionalised copolymers display only a limited solubility in water. As cleavage of disulfides by GSH is reversible, the high concentration of thiols in D50% + CA micellar cores might have favoured the reformation of disulfide-type cross-links, which could explain the slow change in size. D0% treated with GSH showed no sign of swelling, aggregation or disassembly and no apparent change in size. This proved that the CA cross-linked micelles are effectively triggered by the presence of a reducing agent, changing the size distribution of the NPs.

AEE cross-linked NPs (D50% + AEE) were treated under acidic conditions to assess their pH-responsive behaviour. The acetal linkers were thus cleaved into alcohols, which led to a response. The NPs were incubated in water at pH 2 for 4 weeks and their size evolution was monitored by DLS over this period (Fig. 4D). The D50% + AEE micelles exhibited a steady increase of their size from 55 nm at *t*_0_ to 205 nm after 4 weeks, demonstrating their responsiveness to an acid stimulus. On the other hand, only a slight change in size was observed for the unreactive non-cross-linked NPs, D0%, from 100 nm to 110 nm, confirming the responsivity conferred by the AEE moiety.

With the aim of circumventing the slow rate of response in water, the acid-triggered cleavage of D50% + AEE NPs was also performed in THF. The size change was hypothesised to be much faster as a consequence of the better solubility of the core in THF relatively to water. First, the D50% + AEE NPs suspension was reacted with 10 mM HCl in THF, which led to a fast size change of the NPs as evidenced by DLS (Fig. 4E). However, the response time frame, monitored every 10 minutes by DLS, was shorter than the time between the measurement points. There, the size of the nanoparticles even begins to decrease after the rapid initial increase. This observation alludes to potential disassembly of the nanostructures although complete disassembly was not observed under our experimental conditions. To slow down the process, a concentration of 100 μM of HCl in THF was employed resulting in expectedly slower size change (Fig. 4F). In 3 h, the size monitored by DLS increased from 20 nm to 120 nm where it reached a plateau as a result of the swelling of the micelles. This monitoring established that the NiCCo-PISA micelles were indeed pH-sensitive.

## 3. Conclusions

Nano-objects with cross-linked helical cores were readily achieved by NiCCo-PISA, followed by a straightforward PPM methodology involving the reaction between pentafluorophenyl ester-based units and various diamine molecules. The stability of the cross-linked micelles was confirmed by DLS measurements in THF, while the preservation of the helicity was confirmed by CD spectroscopy. NPs that were core cross-linked by cystamine exhibited redox-triggered response, which could be monitored by DLS in water, whereas NPs modified with the acetal-containing cross-linker were found to respond to changes in pH, as evidenced by DLS. Although complete disassembly of nanostructures was not observed in this study, we are actively investigating this process. Nevertheless, changes in core polarity could possibly still facilitate efficient delivery of payload from within the particles. Thus, this study expands the potential of NiCCo-PISA towards applications of the resulting smart nanomaterials in drug delivery and in sensors, or as nanoreactors where the chiral core could be leveraged for enantioselectivity.

## Author contributions

S. J. and J. C. F. conceived this study. S. J. performed the experiments and analysed the data. S. V. obtained the TEM images. D. T., A. P. D. and R. K. O. obtained the funding for this research project. S. J. wrote the original draft. J. C. F., S. V., D. T., A. P. D. and R. K. O. reviewed and edited the manuscript.

## Conflicts of interest

There are no conflicts to declare.

## Supplementary Material

PY-013-D2PY00397J-s001
