# Supplementary material for: Stimuli-responsive and core cross-linked micelles developed by NiCCo-PISA of helical poly(aryl isocyanide)s
Source: Polym Chem. 2022 Jun 13;13(27):4047–53. doi: 10.1039/d2py00397j (PMC9274662; doi:10.1039/d2py00397j)
Supplement: PY-013-D2PY00397J-s001 [file PY-013-D2PY00397J-s001.pdf]

Electronic Supplementary Material (ESI) for Polymer Chemistry.
This journal is © The Royal Society of Chemistry 2022

Supporting Information

Stimuli-responsive and core cross-linked micelles developed by NiCCo-PISA of helical poly(aryl isocyanide)s

Sètuhn Jimaja, Spyridon Varlas, Jeffrey C. Foster, Daniel Taton, Andrew P. Dove, Rachel K. O'Reilly**

S. Jimaja
Department of Chemistry, University of Warwick, Coventry CV4 7AL, United Kingdom

S. Jimaja, S. Varlas, J. Foster, A. P. Dove, R. K. O'Reilly
School of Chemistry, University of Birmingham, Edgbaston B15 2TT, United Kingdom

S. Jimaja, D. Taton
Laboratoire de Chimie des Polymères Organiques, Université de Bordeaux/CNRS École Nationale Supérieure de Chimie, de Biologie & de Physique, 33607 Cedex Pessac, France

E-mail: a.dove@bham.ac.uk, r.oreilly@bham.ac.uk

Materials

Triethylamine (NEt_3), acetic anhydride, *N*-(2-hydroxyethyl)-phthalimide, 2-methoxy propene, 1,6-hexanediamine, cystamine, glutathione (GSH), chloroform ($CHCl_3$), magnesium sulfate ($MgSO_4$), isopropanol (*i*-PrOH), benzene anhydrous (99.8%) and dimethyl sulfoxide (DMSO) anhydrous (≥99%) were purchased from Sigma-Aldrich. DMSO and benzene were dried over molecular sieves overnight before use. Sodium hydroxide (NaOH), Hydrochloric acid (HCl) solution in THF (1.0 M) and molecular sieves (4 Å) were purchased from Fisher Scientific. Tetrahydrofuran (HPLC grade, THF), hexane, ethyl acetate and methanol (MeOH) were purchased from VWR Chemicals. THF was purified *via* passage through a column of basic alumina prior to use. The PEG-ester, menthyl-ester and pentafluorophenyl-ester aryl isocyanide monomers, and *o*-Tol(dppe)NiCl initiator were synthesized and characterized as reported previously.[1, 2] Formvar-carbon coated (300 mesh) and graphene oxide (GO)-coated (300 mesh) copper grids were purchased from EM Resolutions.

Characterisation techniques

NMR Spectroscopy. ^{1}H NMR and ^{19}F NMR spectra were recorded at 300 MHz or 400 MHz on a Bruker DPX-300 or a Bruker DPX-400 spectrometer, using chloroform-*d* ($CDCl_3$) as the solvent. Chemical shifts of protons are reported as δ in parts per million (ppm) and are relative to $CHCl_3$ at δ = 7.26 ppm.

Size Exclusion Chromatography. Size exclusion chromatography (SEC) analysis was performed on a system composed of an Agilent 1260 Infinity II LC system equipped with an Agilent guard column (PLGel 5 μM, 50 × 7.5 mm) and two Agilent Mixed-C columns (PLGel 5 μM, 300 × 7.5 mm). The mobile phase used was THF (HPLC grade) containing 2% v/v NEt_3 at 40 °C at flow rate of 1.0 $mL \cdot min^{-1}$ (polystyrene (PS) standards were used for calibration, range: 370 - 1044000 Da). Number average molecular weights (M_n), weight average molecular weights (M_w) and dispersities ($Đ_M = M_w/M_n$) were determined using the Agilent GPC/SEC software.

Dynamic Light Scattering. Hydrodynamic diameters (D_H) and size distributions (PD) of nano-objects were determined by dynamic light scattering (DLS) using a Malvern Zetasizer Nano ZS with a 4 mW He-Ne 633 nm laser module operating at 25 °C. Measurements were carried out at an angle of 173° (back scattering), and results were analysed using Malvern DTS v7.03 software. All determinations were repeated 5 times with at least 10 measurements recorded for each run. D_H values were calculated using the Stokes-Einstein equation where particles are assumed to be spherical.

Transmission Electron Microscopy. Dry-state stained transmission electron microscopy (TEM) imaging was performed on a JEOL JEM-1400 microscope operating at an acceleration voltage of 80 kV. All dry-state samples were diluted with dionized water to appropriate analysis concentration and then deposited onto formvar-coated or GO-coated copper grids. After roughly 1 min, excess sample was blotted from the grid and the grid was stained with an aqueous 1 wt% uranyl acetate (UA) solution for 1 min prior to blotting, drying and microscopic analysis.

Circular Dichroism Spectroscopy. Circular dichroism (CD) spectra were recorded on a JASCO J810-150S spectropolarimeter using Quartz cells (path length 1 mm, 170 - 2000 nm) from Hellma. For the spectra range 650-450 nm, the following parameters were used: bandwidth 5 nm, data pitch 0.1 nm, scanning speed 100 $nm \cdot s^{-1}$, D.I.T. 4 s. For the spectra range 650-200 nm, the following parameters were employed: bandwidth 5 nm, data pitch 0.2 nm, scanning speed 200 $nm \cdot s^{-1}$, D.I.T. 1 s. 3 accumulations were taken.

Synthetic procedures

General procedure for NiCCo-PISA

The block copolymer was synthesized following a procedure reported previously.[2] In a 7 mL vial, under inert atmosphere, the PAIC monomer in DMSO (0.5 mL) was added to the *o*-Tol(dppe)NiCl initiator (121 μL, 17.5 mg·mL^{-1} in DMSO) and stirred for 2 min. A solution of the MAIC and FAIC monomers in DMSO was then added and the reaction mixture was stirred for an additional 2 min before opening to atmosphere. The reaction's solids weight content was 50 mg·mL^{-1}.

Table S1. Quantities used for the copolymerisation of D0% and D50%.

Polymer	PAIC (mg)	MAIC (mg)	FAIC (mg)	DMSO (mL)
**D0%**	59.0	30.5	-	1.83
**D50%**	59.0	15.2	17.1	1.87

Table S2. Characterisation of **D0%** and **D50%**.

Polymer	D_{DLS} (nm)[a]	$M_{n,SEC}$ (kDa)[b]	$Đ_M$ [b]	CD_{360} (mdeg)[c]
**D0%**	20 (0.18)	11.2	1.24	14
**D50%**	20 (0.25)	10.5	1.34	8.7

[a] Particle size measured by DLS in DMSO with PDI in parenthesis. [b] Determined by SEC (THF + 2% v/v NEt_3) using PS standards. [c] CD (THF, 0.5 mg·mL^{-1}) signal at λ = 360 nm. [d] Molecular weight distribution is outside the calibration range

2-[1-(2-Amino-ethoxy)-1-methyl-ethoxy]-ethylamine (AEE) synthesis

AEE was prepared in accordance with previously reported synthetic methods.[3]

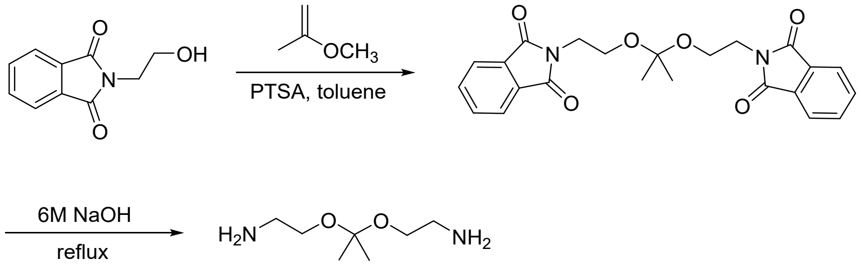

Scheme S1. 2-[1-(2-Amino-ethoxy)-1-methyl-ethoxy]-ethylamine synthesis

In a 500 round-bottom flask, *N*-(2-hydroxyethyl)-phthalimide (12 g, 65 mmol, 1.0 equiv.) was dissolved in dry benzene (200 mL). After the solution was cooled to 0 °C, 2-methoxy propene (6.5 mL, 68 mmol, 1.1 equiv.) and *p*-toluenesulfonic acid (PTSA, 120 mg, 0.010 equiv.) were carefully added to the solution. The reaction mixture was then stirred for 1 h at 0 °C. Then, the solution was dried *in vacuo* before acetic anhydride (7.5 mL) and Et_3N (15 mL) were added to the residue and the resulting suspension was stirred overnight. The crude product was

precipitated in hexane (50 mL) before recrystallisation in ethyl acetate (200 mL) twice which gave the product as a yellow solid.

In a 100 mL round bottom flask, 2,2'-((propane-2,2-diylbis(oxy))bis(ethane-2,1-diyl))bis(isoindoline-1,3-dione) was solubilized in NaOH (6 M, 50 mL) and heated at reflux overnight. The reaction mixture was extracted with $CHCl_3$/*i*-PrOH (1/1, v/v, 3 × 50 mL), the combined organic layers were dried on $MgSO_4$, filtered and concentrated *in vacuo*. The crude product was washed with hexane which yielded the product as a yellow oil. ^{1}H NMR (400 MHz, $CDCl_3$): δ (ppm) 3.46 (t, $^3J_{H-H}$ = 5.4 Hz, 4H, CH_2NH$_2$), 2.84 (t, $^3J_{H-H}$ = 5.3 Hz, 4H, OCH_2), 1.70 (bs, 4H, NH_2), 1.37 (s, 6H, CH_3). ^{13}C NMR (100 MHz, $CDCl_3$): δ (ppm) 99.6 (O*C*O), 62.7 (O*C*H$_2$), 42.0 (*C*H$_2$NH$_2$), 24.8 (*C*H$_3$). Characterization was consistent with that reported previously.[3]

General procedure for the post-polymerization cross-linking of the NiCCo-PISA micelles

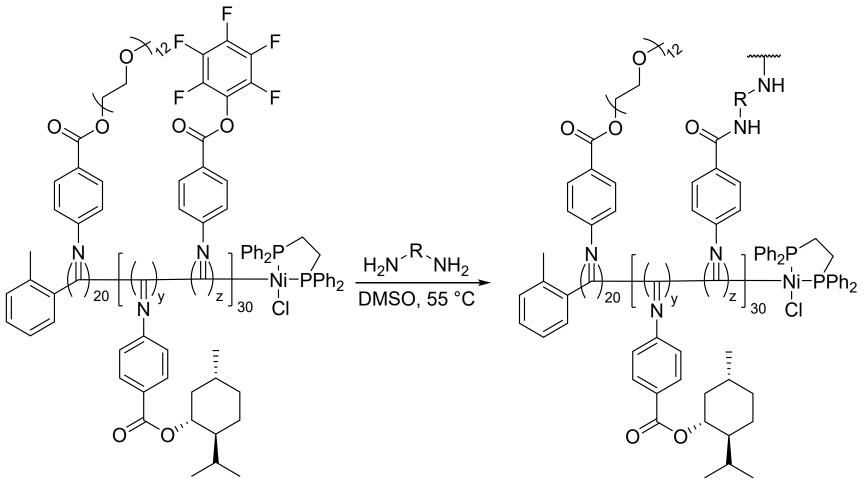

Scheme S2. Post-polymerisation cross-linking of the NiCCo-PISA micelles with diamine nucleophiles.

In a 7 mL vial, a solution of the NiCCo-PISA nano-objects was diluted with DMSO to 10 mg·mL^{-1} and the nucleophile was added (0.60 equiv.). The reaction mixture was stirred for 72 h at 55 °C. The resulting solution was analysed by DLS then, dialysed against water (6-8 kDa cut-off) before analysis by DLS. The solution was freeze-dried and precipitated in hexane from THF before analysis by FT-IR spectroscopy then, dissolved in THF for CD spectroscopy.

General procedure for the monitoring of the cross-linked particles' stimuli-responsiveness

A solution that contained 0.20 $mg \cdot mL^{-1}$ of particles in water or THF was treated with glutathione (GSH) or HCl to obtain the desired concentration. The evolution of the nanostructures' size was monitored by DLS.

Characterisation

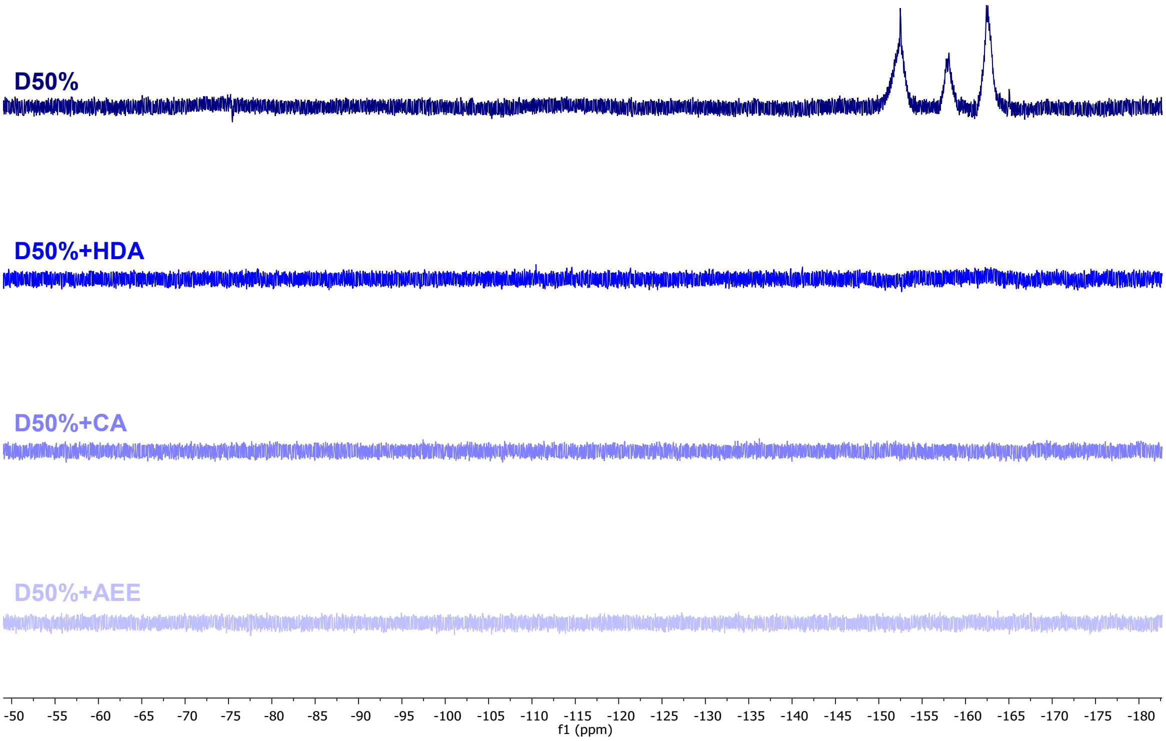

Figure S1. ^{19}F NMR spectra of **D50%**, **D50%+HDA**, **D50%+CA** and **D50%+AEE** in $CDCl_3$ (377 MHz, 298 K).

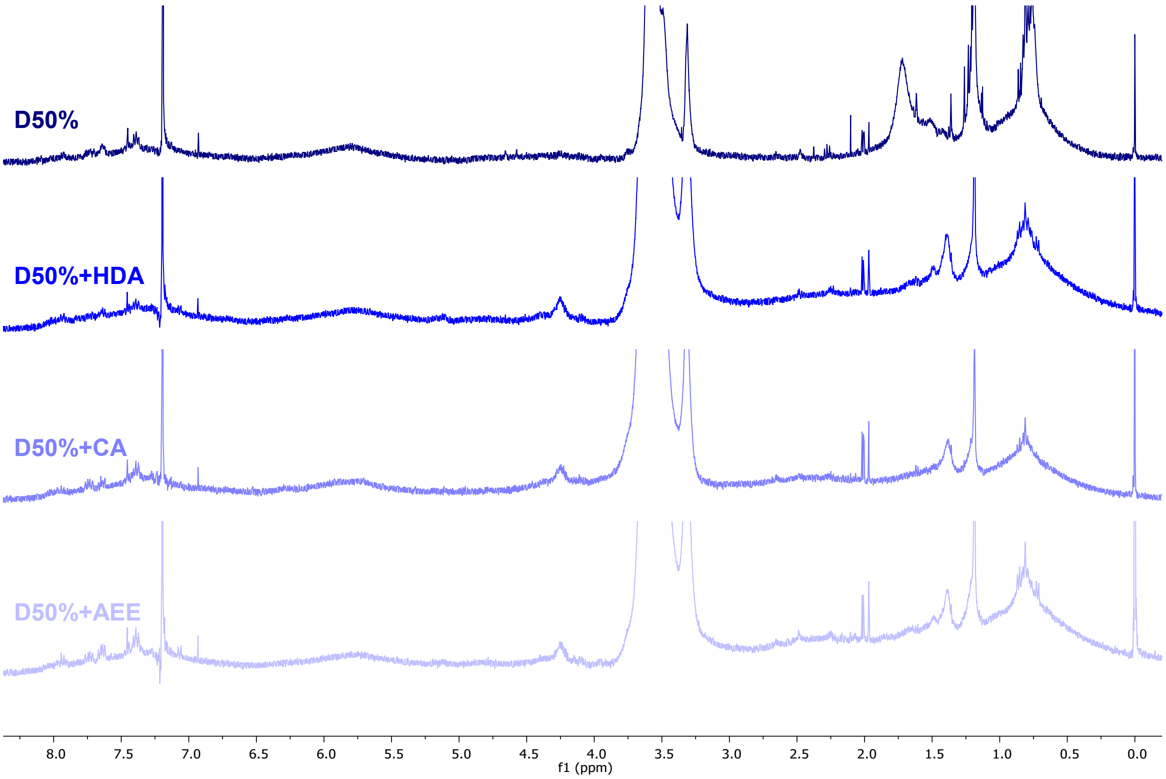

Figure S2. ^{1}H NMR spectra of **D50%**, **D50%+HDA**, **D50%+CA** and **D50%+AEE** in $CDCl_3$ (400 MHz, 298 K).

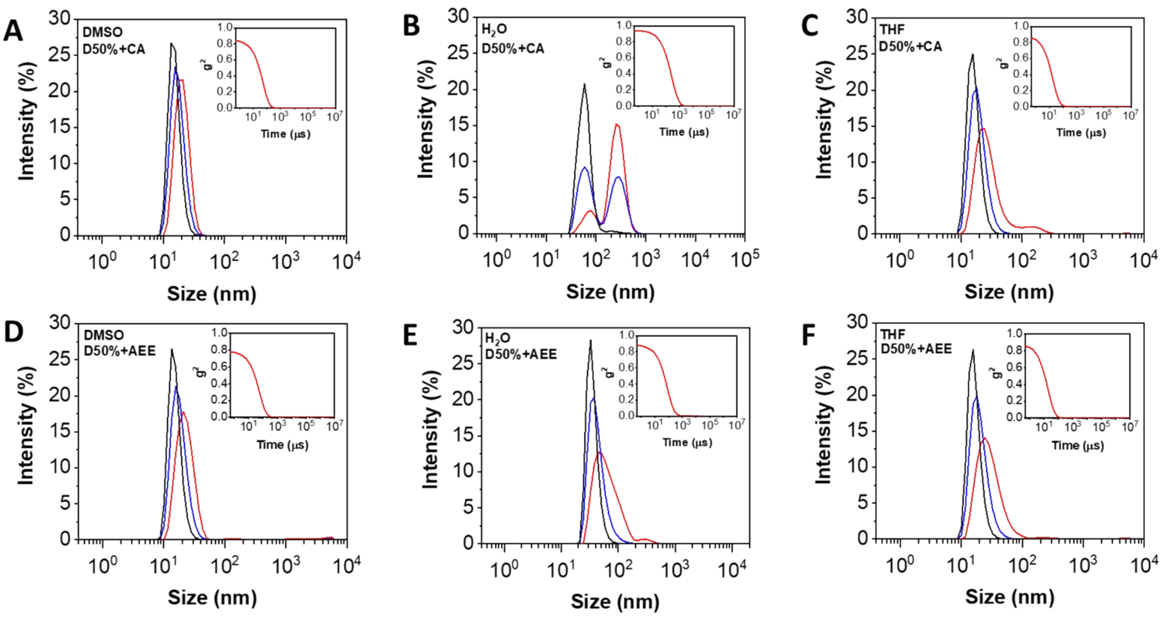

Figure S3. Size distributions of **D50%+CA** in (A) DMSO, (B) H_2O and (C) THF, and **D50%+AEE** in (D) DMSO, (E) H_2O and (F) THF obtained by DLS. The intensity (red line), volume (blue line) and number (black line) distributions are displayed. The insets show the corresponding correlograms in each case.

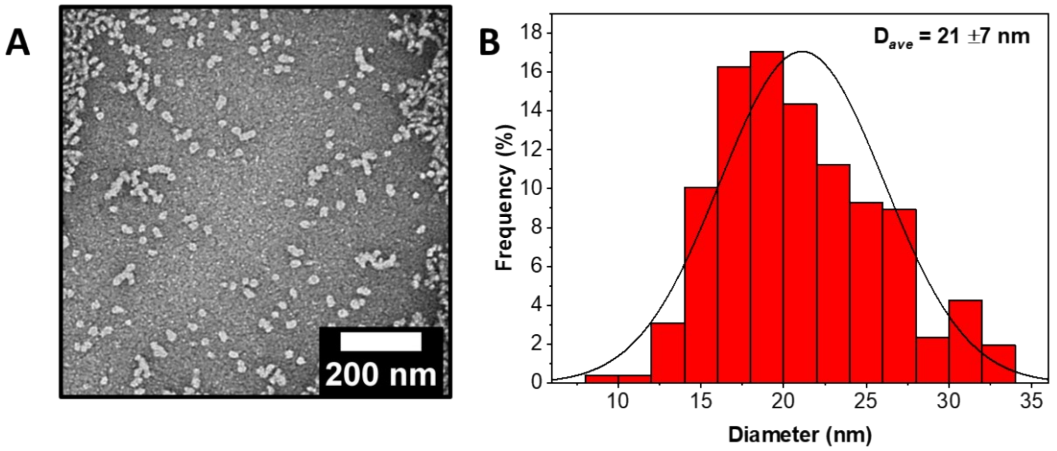

Figure S4. (A) Dry-state TEM image of **D0%**. (B) Histogram of spherical particles' size distribution measured from particle analysis of TEM images of 250 particles.

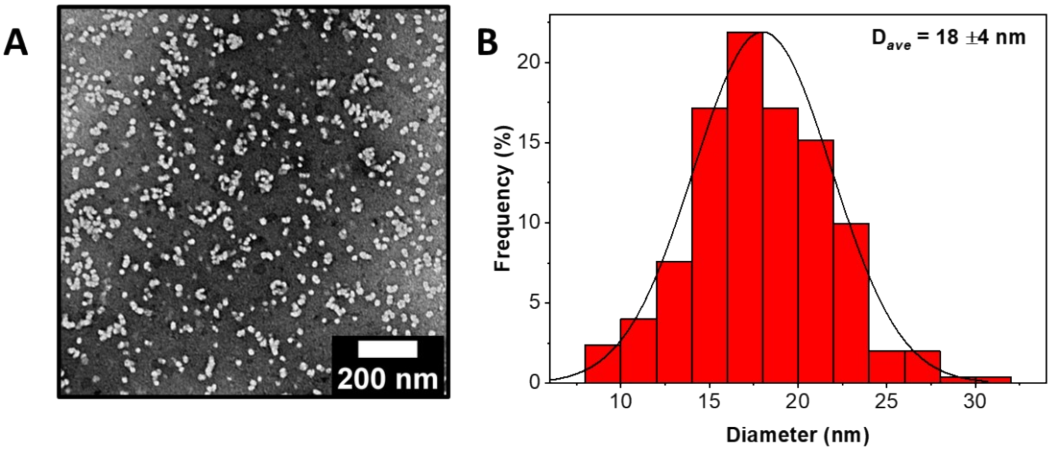

Figure S5. (A) Dry-state TEM image of **D50%**. (B) Histogram of spherical particles' size distribution measured from particle analysis of TEM images of 250 particles.

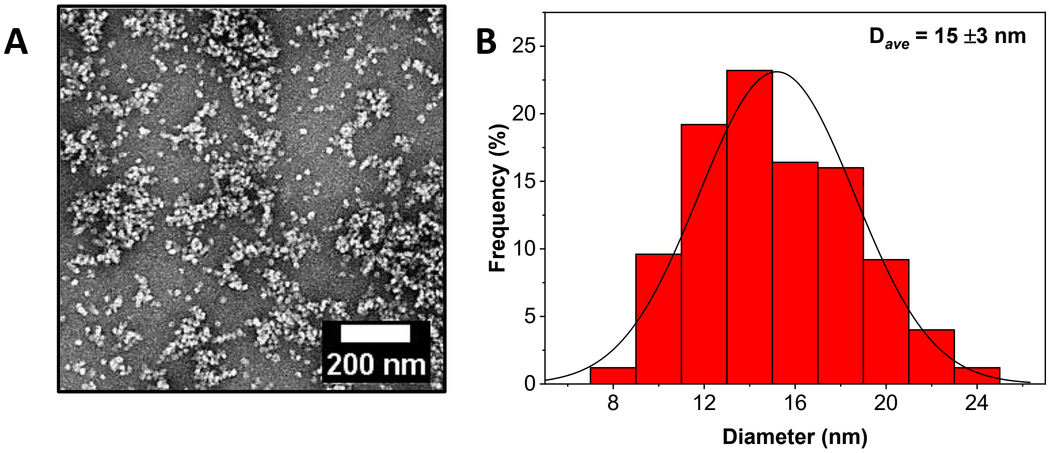

Figure S6. (A) Dry-state TEM image of **D50%+HDA**. (B) Histogram of spherical particles' size distribution measured from particle analysis of TEM images of 250 particles.

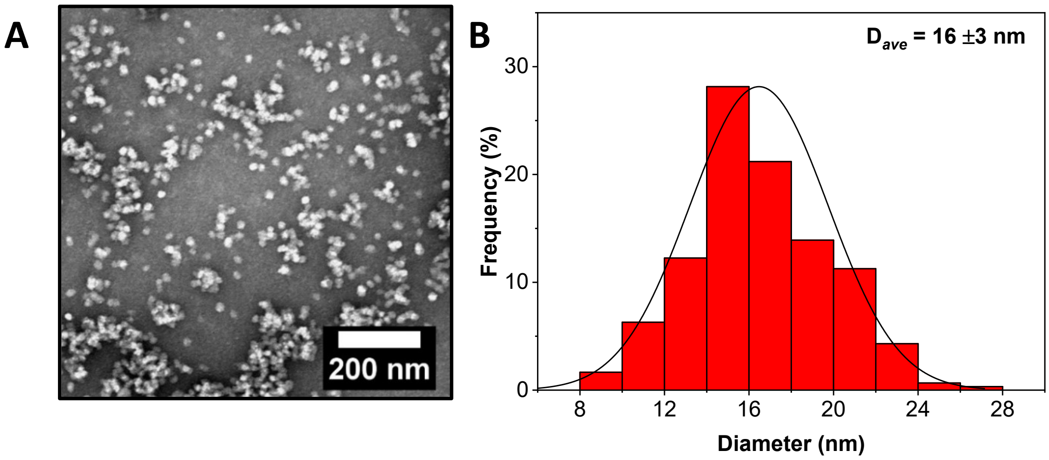

Figure S7. (A) Dry-state TEM image of **D50%+CA**. (B) Histogram of spherical particles' size distribution measured from particle analysis of TEM images of 300 particles.

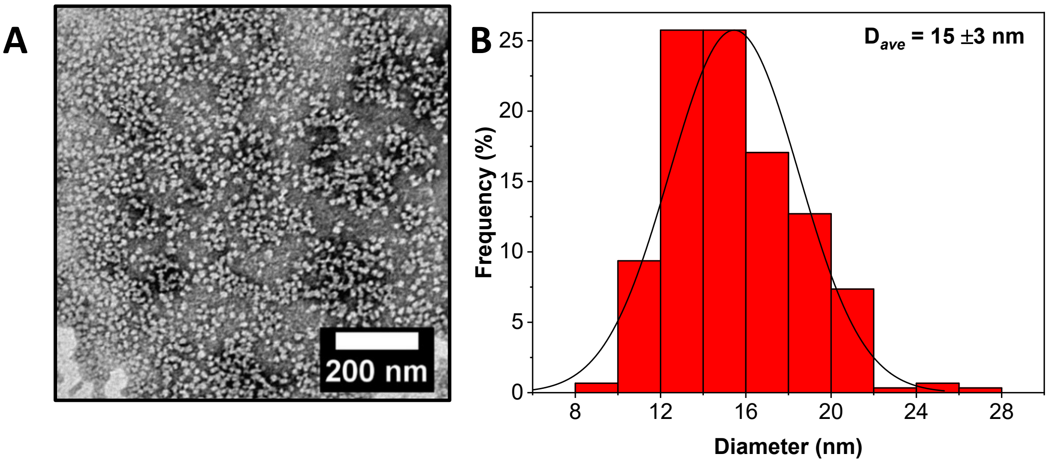

Figure S8. (A) Dry-state TEM image of **D50%+AEE**. (B) Histogram of spherical particles' size distribution measured from particle analysis of TEM images of 300 particles.

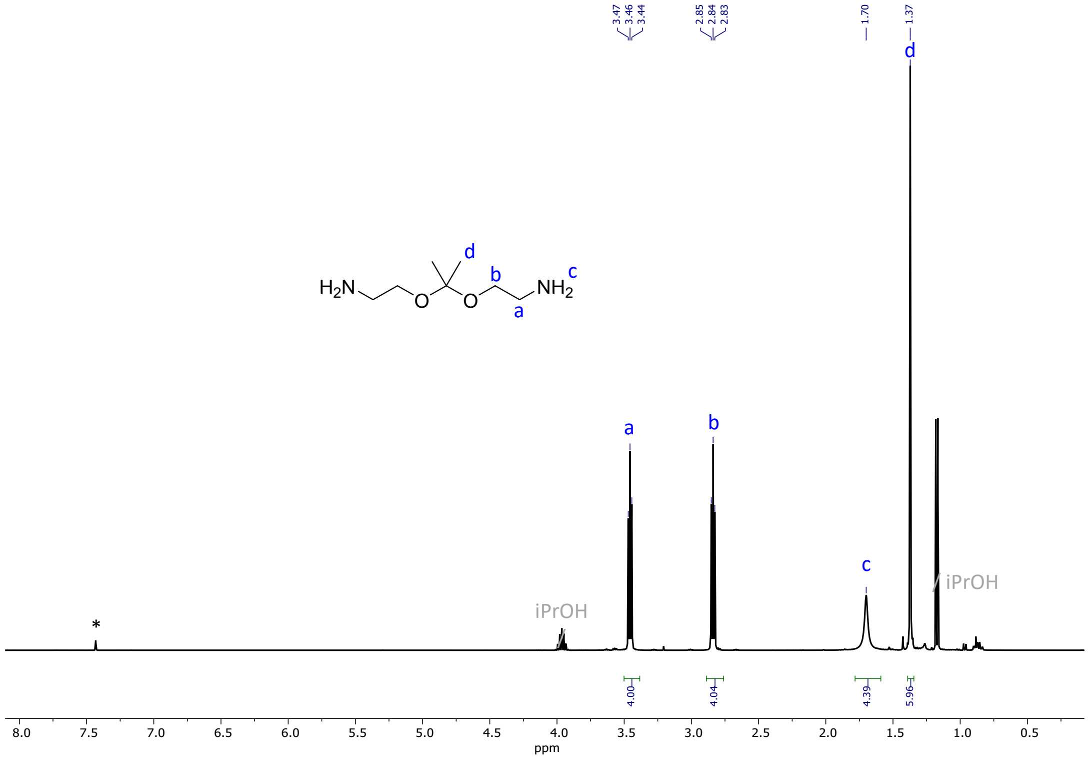

Figure S9. ^{1}H NMR of AEE in $CDCl_3$ (400 MHz, 298 K)

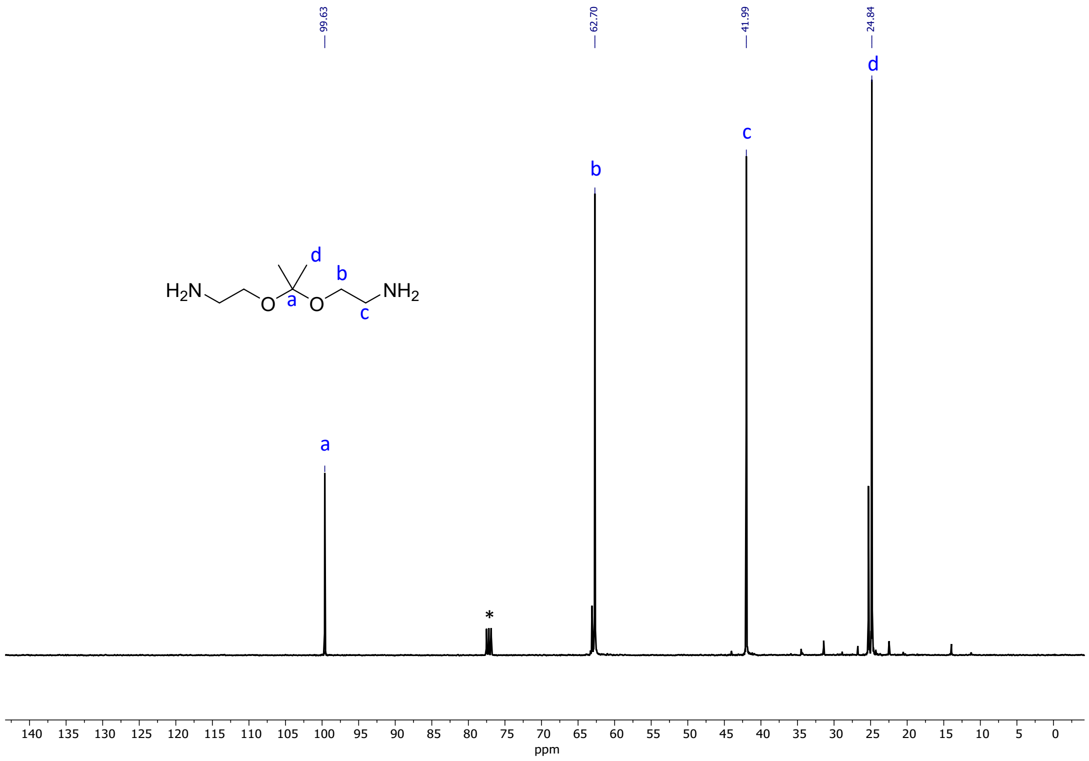

Figure S10. ^{13}C NMR of **AEE** in $CDCl_3$ (100 MHz, 298 K)

References

1. Jimaja, S.; Varlas, S.; Xie, Y.; Foster, J. C.; Taton, D.; Dove, A. P.; O'Reilly, R. K., Nickel-Catalyzed Coordination Polymerization-Induced Self-Assembly of Helical Poly(aryl isocyanide)s. *ACS Macro Lett.* **2020,** *9* (2), 226-232.

2. Jimaja, S.; Xie, Y.; Foster, J. C.; Taton, D.; Dove, A. P.; O'Reilly, R. K., Functional nanostructures by NiCCo-PISA of helical poly(aryl isocyanide) copolymers. *Polym. Chem.* **2021,** *12* (1), 105-112.

3. Paramonov, S. E.; Bachelder, E. M.; Beaudette, T. T.; Standley, S. M.; Lee, C. C.; Dashe, J.; Fréchet, J. M. J., Fully Acid-Degradable Biocompatible Polyacetal Microparticles for Drug Delivery. *Bioconjugate Chem.* **2008,** *19* (4), 911-919.
